# Screening for ADHD-Related Symptoms in Preschoolers Should Be Considered—Results From a Representative Sample of 5-Year-Olds From a German Metropolitan Region

**DOI:** 10.3389/fpsyt.2018.00612

**Published:** 2018-11-20

**Authors:** Konstantin Mechler, Thomas Krömer, Michael Landauer, Ralf W. Dittmann, Alexander Häge

**Affiliations:** ^1^Pediatric Psychopharmacology, Department of Child and Adolescent Psychiatry and Psychotherapy, Central Institute of Mental Health, Medical Faculty Mannheim, University of Heidelberg, Mannheim, Germany; ^2^Practice of Child and Adolescent Psychiatry and Psychotherapy, Hamburg, Germany

**Keywords:** ADHD, attention, preschool, screening, epidemiology, gender

## Abstract

**Background:** Early assessment and intervention are crucial to alleviate symptoms and prevent long-term negative outcomes in children suffering from Attention-deficit/hyperactivity disorder (ADHD). In Germany, at present, no standardized screening for ADHD is routinely administered. This study aims to evaluate a potential screening measure in a study population that is representative for a primary school entrance exam population in a German metropolitan region.

**Methods:** Based on various socio-demographic variables, a sample of *n* = 500 5-year-old children (58% boys, 42% girls), representative of a primary school entrance exam population from a German metropolitan region, was selected. Their parents completed a written survey consisting of the CBCL and a brief screening tool for ADHD symptomatology based on the DISYPS-II questionnaire. Demographic data were also collected.

**Results:** The subscale “Attention problems” of the CBCL/4-18 showed results in the clinical range for *n* = 10 (2%) participants. The ADHD screening identified *n* = 23 (4.6%) participants as suspect of having ADHD with a statistically significant gender difference (*n* = 17 boys vs. *n* = 6 girls, *p* = 0.03). In *n* = 5 (1%) participants, all boys, both CBCL/4-18 and the ADHD screening were indicative of ADHD.

**Conclusions:** Results indicate that screening for ADHD in this population may be both feasible and reasonable given the high prevalence and chronic nature of this disorder and the benefit of an early initiation of treatment. Results match previously reported figures for prevalence of ADHD-related symptoms and gender differences in preschool and older pediatric populations and thus do not support the hypothesis that the prevalence of ADHD in a metropolitan region is significantly higher than in other regions.

## Introduction

Attention-deficit/hyperactivity disorder (ADHD) is characterized by impulsivity, hyperactivity and inattention (1). It is one of the most common psychiatric disorder in children world-wide with a prevalence of ~5% in school-aged children (2–4). Research has identified genetic factors as a main cause for ADHD (5). Yet, etiology remains complex and gene-environment interactions contribute to the overall risk for an individual to develop ADHD, as in most psychiatric disorders (6, 7). ADHD is associated with impairments in (social) functioning and reduced health-related quality of life (8). One or more psychiatric comorbidities, such as affective disorders, sleep disorders, dyslexia, enuresis, tic disorders, conduct disorder, and substance use disorder are present in ~75% of patients with ADHD (9–13). ADHD as a chronic psychiatric disorder has also been related with impairments along the lifespan. Compared to healthy controls, patients with ADHD suffer from, e.g., higher rates of suspensions from school, more than twice as many car accidents, three to five times as many separations/divorces, and a significantly higher risk for an earlier death (6, 9, 14–19). Therefore, early assessment and intervention are crucial to prevent such outcomes in children suffering from ADHD.

Regarding early assessment of ADHD, useful and valid screening tools should be available and routinely administered to children, ideally at the primary school entrance level. The main objective of this study is to evaluate such a measure in a study population that is representative for a real-world primary school entrance exam population in a German metropolitan region. In Germany, at present, no such standardized screening for ADHD is routinely administered.

## Materials and methods

Parents of 5-year-old preschool children from the German metropolitan region Hamburg were asked to complete a written survey. Five hundred families were selected based on the following socio-demographic variables in order to generate a sample representative of this metropolitan region: number of children, mother's marital status, mother's, and father's work status and position. Identification and selection of parents and participants as well as conduction of the written survey were performed by a private survey institute and financially supported by Lilly Deutschland GmbH (Eli Lilly & Co., Germany). Inclusion criteria were: 5;0–5;11 years of age at the time of survey, sufficient level of understanding of the nature and the content of the study by parents, general agreement to potentially be invited to a personal examination by one of our research team's child and adolescent psychiatrists and to be asked to complete a follow-up survey at a later time. Main exclusion criteria were: serious unstable illnesses, non-agreement to further investigation, and previous treatment for ADHD. The latter was chosen in line with previous, similar studies (20) and in order to create a sample representative for a primary school entrance exam population in a real-world setting as children with a previous treatment for ADHD are already professionally cared for and would not benefit from a screening procedure. Other disorders or treatments in any form were not part of the exclusion criteria. The study's protocol was approved by the Ethical Commission of the Medical Association Hamburg, Germany. After participation, families received a small thank-you gift, e.g., flowers, with a value of ~€ 5. The survey consisted of one general child psychopathology questionnaire, one ADHD-specific screening instrument, as well as a number of additional questions assessing psychosocial status and demographic variables:

### Child behavior checklist CBCL/4-18

The CBCL/4-18 is a questionnaire for parents of children aged 4–18 years acquiring information on competencies and problems of children (21) and has been used extensively in international clinical practice and research. By use of 118 items, internalizing (i.e., anxious, depressive, and overcontrolled) as well as externalizing (i.e., aggressive, hyperactive, noncompliant, and undercontrolled) behaviors are assessed and three main scales (Total Problems, Internalizing Problems, and Externalizing Problems), eight empirically based syndrome scales (Aggressive Behavior, Anxious/Depressed, Attention Problems, Rule-Breaking Behavior, Somatic Complaints, Social Problems, Thought Problems, and Withdrawn/Depressed), and four competence scales (Total Competence, Activities, Social, and School) are calculated. Results are then interpreted as lying in the normal (*T* score < 67), borderline (*T* 67–70), or clinical range (*T* score >70). For this study in preschool children, two items were changed to address behavior at kindergarten rather than at school (items 23 and 30). Additionally, two items regarding school were dismissed (items 61 and 101) resulting in a number of total items of 116.

### ADHD screening

**Figure 1:**
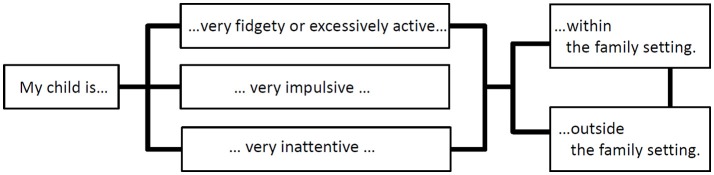
Concept of the parent-rated ADHD screening instrument used in this study assessing three core symptom domains of ADHD within and outside of the home/family setting based on the first three items of the DISYPS-II parent-rated questionnaire for ADHD in 3–6 year old children (23).

A short and easy to complete questionnaire asking parents about core symptomatology of ADHD within and outside of the home/family setting was used for this study (22). This so-called “ADHD sheet” (German: “ADHS-Bogen”) is based on the first three items of the DISYPS-II parent-rated questionnaire for ADHD in 3–6 year old children [see Figure 1; (22)]. The DISYPS-II is a German-language diagnostic questionnaire-based system for the assessment of relevant disorders in child and adolescent psychiatry according to ICD-10 and DSM-IV criteria. The “ADHD sheet” uses a 4-point Likert scale (0: “not at all” −3: “markedly”) to assess severity of the symptom dimensions in question. In line with the ICD-10 diagnostic criteria for ADHD, the cut-off for a child to be suspect of having ADHD is defined as a score of ≥2 in both settings (within/outside of home/family) for the hyperactive and impulsive domain combined and/or the inattentive domain.

### Additional variables

Further information to be collected from the participants was, among others, data on previous and current treatments and medications and various demographic data.

### Statistical analyses

Demographics were summarized descriptively using participant count and percentages of the total population. Furthermore, demographic variables (e.g., gender) were compared using Fisher's exact tests as participant counts were to be expected lower than *n* = 5 for several items. *P*-values reported are two-sided. *P*-values of 0.05 or less were deemed statistically significant. Data were collected in Microsoft® Office Excel. All analyses were performed with IBM® SPSS® Statistics Version 22.

## Results

### Characteristics of study population

Parents of *n* = 260 boys (52%) and *n* = 240 girls (48%), all between 5;0 and 5;11 years old and selected in order to create a representative sample, took part in this study. The questionnaire was completed by mothers in *n* = 450 (90%), by fathers in *n* = 41 (8.2%), and by other family members in *n* = 9 (1.8%) of cases.

The study population showed a low exposure to psychiatric/psychological treatment. At the time of survey, *n* = 1 (0.2%) participant was treated by a child and adolescent psychiatrist, *n* = 8 (1.6%) by a psychologist. Parents reported that their children had been treated because of mental or conduct problems in the past (>6 months ago) in *n* = 16 (3.2%), and recently or currently in *n* = 31 (6.2%) cases. None of the participants received psychotropic medication (antidepressants, methylphenidate, antipsychotics or benzodiazepines) at the time of survey. No statistically significant gender differences were found for all characteristics describing the study population (see Table 1).

**Table 1:** Characteristics of the study population.

	**Total (*****n*** = **500)**		**Male (*****n*** = **260)**		**Female (*****n*** = **240)**		
	***n***	**%**	***n***	**%**	***n***	**%**	***p*-value (male vs. female)**
Attends a kindergarten or comparable institution	348	69.6	179	68.8	169	70.4	*0.77*
Special education kindergarten	2	0.4	2	0.8	0	0	*0.50*
Other institution	36	7.2	17	6.5	19	7.9	*0.61*
Does not attend any institution	110	22	61	23.5	49	20.4	*0.45*
Treatment because of mental or behavioral problems more than 6 months ago	16	3.2	11	4.2	5	2.1	*0.21*
Treatment because of mental or conduct problems within last 6 months or ongoing	31	6.2	21	8.1	10	4.2	*0.09*
Treated by child psychiatrist	1	0.2	1	0.4	0	0	*1.0*
Treated by psychologist	8	1.6	6	2.3	2	0.8	*0.29*
Currently on medication (e.g., antiallergics, antibiotics, nonsteroidal anti-inflammatory drugs)	30	6	14	5.4	16	6.7	*0.58*
Antidepressants	0	0	0	0	0	0	*1.00*
Psychostimulants (e.g., methylphenidate)	0	0	0	0	0	0	*1.00*
Antipsychotics	0	0	0	0	0	0	*1.00*
Benzodiazepines	0	0	0	0	0	0	*1.00*

*p-values from Fisher's exact test*.

### Child behavior checklist CBCL/4-18

Most relevant for the aim of this study, the subscale “Attention problems” of the CBCL/4-18 showed results in the clinical range for *n* = 10 (2%) cases. The other subscales showed the following results, sorted from highest to lowest percentage: aggressive behavior (*n* = 19; 3.8%), rule-breaking behavior (*n* = 17; 3.4%), anxious/depressed (*n* = 19; 3%), thought problems (*n* = 14; 2.8%), social problems (*n* = 12; 2.4%), somatic complaints (*n* = 12; 2.4%), withdrawn (*n* = 7; 1.4%). Statistically significant gender differences were not present in any of the CBCL scores. For a full depiction of CBCL/4-18 results, see Table 2.

**Table 2:** CBCL/4-18 scores within clinical range.

	**Total (*****n*** = **500)**		**Male (*****n*** = **260)**		**Female (*****n*** = **240)**		
	***n***	**%**	***n***	**%**	***n***	**%**	***p*-value (male vs. female)**
Withdrawn	7	1.4	5	1.9	2	0.8	*0.45*
Somatic complaints	12	2.4	8	3.1	4	1.7	*0.39*
Anxious/depressed	15	3	4	1.5	11	4.6	*0.06*
Social problems	12	2.4	9	3.5	3	1.3	*0.15*
Thought problems	14	2.8	8	3.1	6	2.6	*0.79*
Attention problems	10	2	8	3.1	2	0.8	*0.11*
Rule-breaking behavior	17	3.4	8	3.1	9	3.8	*0.81*
Aggressive behavior	19	3.8	11	4.2	8	3.3	*0.65*
Internalizing problems	54	10.8	27	10.4	27	11.3	*0.77*
Externalizing problems	70	14	32	12.3	38	15.8	*0.30*
Total problems	63	12.6	34	13.1	29	12.1	*0.79*

*p-values from Fisher's exact test*.

### ADHD screening (“ADHD sheet”)

According to the cut-off described in the methods section, *n* = 23 (4.6%) participants were suspect of having ADHD. This group consisted of *n* = 17 (6.5%) boys and *n* = 6 (2.5%) girls. This gender difference was statistically significant (*p* = 0.03, Fisher's exact test).

### CBCL and ADHD screening combined

For *n* = 5 (1%) of the children, which were all boys, both CBCL/4-18 and the ADHD screening were indicative of ADHD (Table 3). Yet, this gender difference was of trend character and marginally not statistically significant (*p* = 0.06, Fisher's exact test).

**Table 3:** Results from ADHD screening and CBCL/8-14 combined.

	**Total (*****n*** = **500)**		**Male (*****n*** = **260)**		**Female (*****n*** = **240)**		
	***n***	**%**	***n***	**%**	***n***	**%**	***p*-value (male vs. female)**
ADHD screening results suspect of having ADHD	23	4.6	17	6.5	6	2.5	**0.03**
CBCL within clinical range for attention problems	10	2	8	3.1	2	0.83	*0.11*
Both CBCL and ADHS screening positive	5	1	5	1.9	0	0	*0.06*

*p-values from Fisher's exact test. Statistically significant p-values (≤ 0.05) in bold*.

## Discussion

To the authors' knowledge, this is the first study to assess ADHD-related symptomatology in a large sample representative of a primary school entrance exam population in a German metropolitan region. Depending on the instrument applied, resulting rates for participants suspect of having ADHD were 2% (CBCL), 4.6% (ADHD screening), and 1% (CBCL and ADHD screening combined). Prevalence rates between 2% and 9.6% have previously been reported for ADHD in preschool children (23–25). For purposes similar to this study, the Strengths and Difficulties Questionnaire [SDQ; (26)] has previously been found to be not ideal but acceptable for use in preschool populations (27). It has also been investigated as a screening instrument for ADHD specifically in preschool populations (20, 28, 29). Additionally, a Danish cohort study in *n* = 3,501 children (5–7 years old) found a prevalence of problems of hyperactivity/inattention in 0.7% with a gender ration boys vs. girls of 2:1 (30). These results are comparable to this study's results, even when keeping in mind methodological (previous treatment for ADHD excluded in this study) and geographical (lower rates of ADHD prevalence in Scandinavian countries) differences. The Danish cohort study also found comparable results regarding prevalences of conduct (3 vs. 3.4% in this study) and emotional problems (1.5 vs. 3% in this study).

While data on preschool children is limited, evidence for the prevalence of ADHD in school-age children around 5% is extensive (3, 4). Depending on the instrument, the rates for children suspect of having ADHD in this study either match these figures or appear lower.

It is important to note that the questionnaires used in this study cannot provide or be a substitute for a full child and adolescent psychiatric diagnostic assessment. Yet, for purposes of a routine screening of preschool children the “ADHD sheet” used in this study may be useful as it can easily and quickly be administered, yet follows the diagnostic criteria of ICD-10. Taking into account the relatively high prevalence of ADHD, its probable impairments and the fact that symptoms might not be apparent in a routine physical exam (in contrast to other disorders such as autism), a screening for ADHD could prove useful. If a child was screened positive and thus ADHD may be present, a full diagnostic workup could be performed by a child psychiatrist in a second step. Such a process could identify children with ADHD more reliably before symptoms and/or development of comorbidities may lead to severe negative outcomes, and could allow for adequate treatment. For this reason it would also prove to be cost-effective by avoiding later healthcare and social welfare cost. Nevertheless, these positive aspects must be carefully weighed against the potential negative effects related to a general screening for 5-year-old children. Most importantly, a screening would also result in false positive and false negative cases. For false positive cases, the result of the screening could prove stressful for the parents and families affected, and may cause them to see their children in a “new light”. It may appear to the parents that their child has more problems than before and this could be a negative factor for the children's healthy development. False negative cases would not benefit from the screening.

A diagnostic assessment for ADHD at the school entry age needs to be performed very carefully considering the developmental stage of each individual child. This is especially important in the population of preschool children, which shows a physiologically wide distribution for attention abilities, impulsivity, and hyperactivity. Further studies could further investigate whether the positive effects of routinely performed early screening for ADHD exceeds its potential negative effects.

Since this study investigated screening measures and did not include a full diagnostic assessment, the actual prevalence of ADHD in the study popoulation might be even lower. Consequently, these results are not in support of the hypothesis that the prevalence of ADHD in a metropolitan region is significantly higher than in other regions. If this were the case and environmental factors in a metropolitan region did cause a higher prevalence, one would expect much higher rates of children suspect of having ADHD in this study. Additionally, this set of data seems to further substantiate previous research suggesting that ADHD prevalence is rather robust across geographical regions (3, 31, 32).

CBCL/4-18, by its design, does not specifically assess ADHD symptomatology. Thus, for this study the subscale “Attention Problems” was used as an estimate in combination with the ADHD-specific screening instrument. The combination of data from both instruments led to more refined results with *n* = 5 boys and no girl being suspect of having ADHD. This gender difference may seem obvious, yet did not reach statistical significance (*p* = 0.06, Fisher's exact test). The gender difference, when using the results of the ADHD screening instrument only, was statistically significant (*p* = 0.03, Fisher's exact test).

This study was designed to reflect the real world setting as all participants and parents agreed to be possibly invited to a personal examination by one of our research team's child and adolescent psychiatrists and also to be asked to complete a follow-up survey some years after the initial survey. It is planned to perform, analyze and publish a comprehensive clinical investigation of a subgroup identified as suspect of having ADHD in the future.

Regarding early intervention and following the model of gene-environment interaction, the question arises whether environmental factors could be influenced early on to benefit children with a genetic predisposition to develop ADHD. Children growing up in urban areas are exposed to very different environmental conditions than those in rural areas. They may be exposed to more potentially harmful agents from pollution, to a higher number of psychosocial stressors, play less outdoors in natural environments, and consequently may consume more electronic media (33–36). The possible effects of urbanization as a modern phenomenon and its widespread discussion in society, media and science are not met with sufficient scientific evidence to allow for robust statements, especially in ADHD (37). In addition to the main objective, this study aims to provide evidence for testing the hypothesis that the described environmental factors lead to a significantly higher prevalence of ADHD in metropolitan regions. This would show as a higher rate of children suspect of having ADHD in this study's sample representative of a primary school entrance exam population.

## Limitations

This study has several limitations. First, children/families with an earlier ADHD diagnosis or respective treatment were intentionally excluded from this study (cf. Methods). This limits comparability with earlier reported rates from studies investigating prevalence of ADHD. Yet, prevalence of ADHD has previously been investigated elsewhere and was not the focus of this study. Main focus was rather the evaluation of a screening measure which is most adequately done in a sample population without a portion of subjects who have already undergone a diagnostic workup and therapeutic interventions for the disorder in question. This reflects a real-world situation where a child with a diagnosis and treatment of ADHD would not be screened for ADHD. Second, a selection bias may have occurred. A general and widespread problem in clinical research, only willing and interested parents may have participated. Though relatively small, the financial reimbursement may also have acted as an additional incentive to participate. This might have led to a non-representative sample. Yet, in this study, a large sample was recruited and social and demographic variables were taken into account for the final selection of participants in order to create a representative sample. Third, the data relied on parent report, solely. Although the confidential nature of the survey was clearly communicated to the parents, it remains possible that some did not honestly complete all questions due to response bias. In order to eliminate this limitation, a follow-up clinical examination of a subgroup of participants by a child and adolescent psychiatrist is currently under way. Fourth, the instruments applied fulfill only a screening purpose and therefore show clear limitations regarding the diagnostic validity of their results. Fifth, interpretation of these results mandates caution due to the young age of participants and a possible overlap of relevant symptoms with physiological developmental phenomena. Sixth, the families participating in this study were selected from a large database of families by the private survey institute according to the inclusion criteria described above. Unfortunately, the latter was not able to provide the authors with exact figures on how many families did not accept the invitation to participate or were excluded. This data is consequently missing in the analysis and discussion of this study.

## Conclusion

These results from a large sample representative of a primary school entrance exam population in a German metropolitan region indicate that screening for ADHD in this population may be both reasonable and feasible given the high prevalence and chronic nature of this disorder and the benefit of an early initiation of adequate treatment. Furthermore, the results match previously reported figures for prevalence of ADHD-related symptoms and gender differences in preschool and older pediatric populations. Considering the limitations of this study, results do not support a hypothesis that the prevalence of ADHD in a metropolitan region is significantly higher than in other regions. A comprehensive clinical investigation of a subgroup of children identified as suspect of having ADHD has been planned to be performed, analyzed, and published in the future.

## Author contributions

KM performed the statistical analysis, interpreted the data, wrote the manuscript with AH, and approved the final manuscript as submitted. TK conceptualized and designed the study, collected and interpreted the data, critically reviewed the manuscript, and approved the final manuscript as submitted. ML interpreted the data and provided substantial clinical and statistical input, critically reviewed the manuscript, and approved the final manuscript as submitted. RD conceptualized and designed the study, interpreted the data and provided substantial clinical and statistical input, critically reviewed the manuscript, and approved the final manuscript as submitted. AH interpreted the data and provided substantial clinical and statistical input, wrote the manuscript with KM, and approved the final manuscript as submitted.

### Conflict of interest statement

KM has served as investigator in clinical trials conducted by Lundbeck, Shire, Sunovion and Teva, plus in EU FP7 programme and Horizon 2020 funded projects. TK reports no potential conflicts of interest. ML has served as investigator in clinical trials conducted by Lundbeck plus in EU FP7 programme funded projects. RD has received compensation for serving as consultant or speaker, or he or the institution he works for have received research support or royalties from the organizations or companies indicated: EU (FP7 Programme), US National Institute of Mental Health (NIMH), German Federal Ministry of Health/Regulatory Agency (BMG/BfArM), German Federal Ministry of Education and Research (BMBF), German Research Foundation (DFG), Volkswagen Foundation; Boehringer Ingelheim, Ferring, Janssen-Cilag, Lilly, Lundbeck, Otsuka, Servier, Shire, Sunovion/Takeda and Theravance. Dr. Dittmann owns Eli Lilly stock. AH has served as investigator in clinical trials conducted by Janssen-Cilag, Otsuka, Shire, Lundbeck, Sunovion and Teva, plus in EU FP7 programme and Horizon 2020 funded projects. He has received conference travel support and compensation for serving as consultant or speaker by E. Lilly and Shire. The remaining authors declare that the research was conducted in the absence of any commercial or financial relationships that could be construed as a potential conflict of interest.
